# Zika virus inhibits eIF2α-dependent stress granule assembly

**DOI:** 10.1371/journal.pntd.0005775

**Published:** 2017-07-17

**Authors:** Raquel Amorim, Abdelkrim Temzi, Bryan D. Griffin, Andrew J. Mouland

**Affiliations:** 1 Lady Davis Institute at the Jewish General Hospital, Montréal, Québec, Canada; 2 Department of Medicine, Division of Experimental Medicine, McGill University, Montréal, Québec, Canada; 3 Special Pathogens Program, National Microbiology Laboratory, Public Health Agency of Canada, Winnipeg, Manitoba, Canada; 4 Department of Medical Microbiology and Infectious Diseases, University of Manitoba, Winnipeg, Manitoba, Canada; 5 Department of Microbiology and Immunology, McGill University, Montréal, Québec, Canada; Baylor College of Medicine, UNITED STATES

## Abstract

Zika virus (ZIKV), a member of the Flaviviridae family, is the most recent emerging arbovirus with pandemic potential. During infection, viruses trigger the host cell stress response, leading to changes in RNA translation and the assembly of large aggregates of stalled translation preinitiation complexes, termed stress granules (SGs). Several reports demonstrate that flaviviruses modulate the assembly of stress granules (SG). As an emerging pathogen, little is known however about how ZIKV modulates the host cell stress response. In this work, we investigate how ZIKV modulates SG assembly. We demonstrate that ZIKV negatively impacts SG assembly under oxidative stress conditions induced by sodium arsenite (Ars), a treatment that leads to the phosphorylation of eIF2α. By contrast, no measurable difference in SG assembly was observed between mock and ZIKV-infected cells treated with sodium selenite (Se) or Pateamine A (PatA), compounds that trigger eIF2α-independent SG assembly. Interestingly, ZIKV infection markedly impaired the phosphorylation of eIF2α triggered in Ars-treated infected cells, and the abrogation of SG assembly in ZIKV-infected cells is, at least in part, dependent on eIF2α dephosphorylation. These data demonstrate that ZIKV elicits mechanisms to counteract host anti-viral stress responses to promote a cellular environment propitious for viral replication.

## Author summary

Zika virus (ZIKV) is transmitted to humans primarily through mosquito bites, but there have also been cases of sexual, perinatal, and suspected blood transfusion transmission. It has been associated with fetal malformations and neurological disorders in adults. The rising concern about this pathogen led the World Health Organization to declare it as a public health emergency of international concern regarding neurological disorders. There is an urgent global scientific effort underway to better understand ZIKV biology and define interactions that occur between the virus and the host cell. We evaluated how ZIKV infection counteracts the assembly of dynamic aggregates of RNA and proteins called stress granules (SGs). We observed that ZIKV blocks SG assembly induced by sodium arsenite (Ars), but not by sodium selenite or Pateamine A. We demonstrate that this difference is related to the ability of ZIKV to modulate the dephosphorylation of eIF2α via its phosphatase. Our work demonstrates that ZIKV prevents a host stress response in order to maintain a cellular environment propitious for viral replication.

## Introduction

Zika virus (ZIKV) is a positive-sense, single-stranded RNA virus that belongs to the genus Flavivirus of the family Flaviviridae, which also includes yellow fever (YFV), West Nile (WNV), dengue (DENV) and Japanese encephalitis viruses (JEV) [1]. The genome of ZIKV encodes a large polyprotein precursor that is co- and post-translationally processed by viral and cellular proteases into three structural proteins [capsid (C), precursor of membrane (prM), and envelope (E)] and seven nonstructural proteins [(NS1, NS2A, NS2B, NS3, NS4A, NS4B and NS5)] that are involved in virus replication, which takes place in the cytoplasm of the host cell [2]. Like other Flavivirus members, ZIKV relies mainly on arthropods such as mosquitoes or ticks for transmission and thus is classified as an arthropod-borne virus (arbovirus). The main arthropod vectors of ZIKV are Aedes sp. mosquitoes (*A*. *aegypti* or *A*. *albopictus*) [3]. Along with the vector-borne transmission, other routes of ZIKV transmission have been demonstrated, including sexual transmission, transplacental and perinatal transmission and blood transfusion [4], raising the concern about the global spread of the disease.

ZIKV was first isolated from a rhesus monkey in the Zika Forest (Uganda) in 1947 [5]. For more than 50 years, ZIKV was rarely reported to cause disease in humans and was commonly associated with mild illness. In 2007, there was an outbreak in the Federated States of Micronesia [6], followed by outbreaks in French Polynesia in 2013–14, in which severe neurological complications were reported [7]. Since then, ZIKV is considered to be the most recent emerging arbovirus with pandemic potential [8]. In 2015, autochthonous transmission of ZIKV was confirmed in the northeastern region of Brazil [9]. A dramatic increase in reported cases of microcephaly in the affected Brazilian regions suggested an association between ZIKV infection and fetal malformations [10] and neurological disorders in adults, including Guillain-Barré syndrome and meningoencephalitis [11]. In February 2016, the World Health Organization declared a public health emergency of international concern regarding neurological disorders associated with the rapid emergence of ZIKV in Oceania and the Americas [12].

In response to conditions of environmental stress, eukaryotic cells activate kinases (HRI, GCN2, PKR and PERK) that phosphorylate eIF2α (eukaryotic initiation factor 2 alpha) to ease cellular injury or, alternatively, to induce apoptosis. Phosphorylation of eIF2α reduces global translation by impairing the formation of the ternary complex eIF2-GTP-tRNA^Met^, allowing cells to conserve resources and to initiate a reconfiguration of gene expression to effectively manage stress conditions [13]. Protein synthesis arrest triggers the assembly of stress granules (SG), that are large ribonucleoprotein (mRNP) aggregates formed by stalled translation preinitiation complexes [14, 15]. The major components of SG are untranslated mRNAs, eukaryotic translation initiation factors (eIF4E, eIF4G, eIF4A, eIF2), the 40S ribosomal subunit and RNA-binding proteins such as the poly(A) binding protein (PABP), T-cell intracellular antigen 1 (TIA-1), TIA-1-related protein (TIAR), and Ras GTPase activating protein-binding protein 1 (G3BP1) [16]. Distinct cell host processes are interrupted or co-opted during viral infection, leading to the activation of cell stress responses on many levels. SG assembly lowers the cytosolic availability of components of the cellular translation machinery and functions as a platform that connects stress and antiviral innate responses, implying an overall antagonistic relationship between viruses and SGs [17]. In this sense, viruses have evolved a plethora of strategies to guarantee their replication by preventing or blocking SG assembly in infected cells, for example by co-opting RNA granule factors and/or blockage of activation of eIF2α kinases, such as PKR [18].

Cellular stress responses are essential in eliciting immune detection and in the cell’s ability to shut down viral gene expression in response to viral infection. So far, little is known about how ZIKV modulates stress responses in infected cells. Recently, it was shown that ZIKV infection triggers a potent repression of host cell translation initiation, while viral protein synthesis remains unaffected [19]. The interplay between viral replication and the cellular stress response may contribute to the exacerbated pathogenesis seen in the current epidemic. Elucidation of the interaction of viral components with host factors involved in SG assembly will provide new insight into the pathology of ZIKV infection. In this work, we investigated how ZIKV infection modulates SG assembly.

## Methods

### Cells and viruses

Green African monkey kidney (Vero) (ATCC) cells and human osteosarcoma-derived U2OS containing G3BP1-GFP (a kind gift from Dr. Paul Anderson and Nancy Kedersha, Harvard Medical School [20]) cells were maintained at 37°C and 5% CO_2_ atmosphere in Dulbecco’s modified Eagle’s medium (DMEM) supplemented with 10% fetal bovine serum (FBS) (HyClone) and 1% penicillin/streptomycin (Life Technologies). Cell viability was evaluated by trypan blue exclusion cytotoxicity assay [21]

To produce viral stocks, Vero cells were infected with ZIKV strain PRVABC59/2015 at a multiplicity of infection (MOI) of 0.01 and incubated for 3 days at 37°C. Viral supernatants were then harvested, centrifuged at 300 x g for 10 minutes at 4°C and filtered on a 45 μm syringe filter. Viral titers were determined by plaque forming assay using culture media supplemented with carboxymethylcellulose (Sigma) as described previously [22]. A stock with a viral titer of 2 x 10^7^ was used in the experiments.

For immunofluorescence assays, 7.5 x 10^4^ Vero or U2OS cells were seeded on 18 mm diameter coverslips the day prior infection. For Western blotting analysis, 7.5 x 10^4^ Vero or U2OS cells were seeded in each well of a 12-well plate. Then, cells were incubated for 1 hour with ZIKV diluted in DMEM at an MOI of 0.5 [23]. After this period, the viral inoculum was removed by aspiration and cells were incubated in complete culture media for the periods specified in each experiment.

### Labeling and detection of *de novo*-synthesized viral RNA

Vero cells were seeded on 18 mm coverslips and infected as described above. Viral RNA was labeled as described in [24]. Briefly, cells were treated for 30 minutes with 1 μg/mL Actinomycin D (Sigma) to block host cellular transcription. Then, cells were transfected with 10 mM 5-bromourudine 50-triphosphate (BrUTP) (Sigma) using Lipofectamine 2000 reagent (Invitrogen). After 1 hour, cells were fixed and processed for indirect immunofluorescence analysis.

### Drug treatments

Stress was induced using 500 μM sodium Ars (NaAsO_2_; Sigma-Aldrich) for 1 h [24], 300 nM Pateamine A (a kind gift from Jerry Pelletier, McGill University) for 1 h, 1 mM sodium selenite (Na_2_SeO_3_; Sigma-Aldrich) for 2 h and 2mM dithiothreitol (DTT; Invitrogen) for 1 h [25]. The eIF2α-dephosphorylation inhibitors Salubrinal (Sigma-Aldrich) and Sal003 (a kind gift from Colin Crist, McGill University) were used at final concentrations ranging from 5 to 75 μM by the time described in each experiment.

### Antibodies and reagents

Goat anti-TIAR (Santa Cruz Biotechnology) was used for indirect immunofluorescence microscopy at a dilution of 1:500; rabbit anti-eIF4G (Santa Cruz Biotechnologies) was used for indirect immunofluorescence at 1:500; mouse anti-Zika NS1 (BioFront Technologies) was used at 1:500 for indirect immunofluorescence and 1:1,000 for Western blotting; mouse anti-BrUTP (Enzo Life Sciences) was used for indirect immunofluorescence at 1:100; rabbit anti-phospho eIF2α (Ser51) (Cell Signaling Technology) was used for indirect immunofluorescence and 1:500 and for Western blotting at 1:1,000; mouse anti-eIF2α (Cell Signaling Technology) was used for Western blotting at 1:1,000; and mouse anti-actin (Abcam) was used for Western blotting at 1:10,000; rabbit anti-GADD34 (Thermo Fisher Scientific) was used for western blotting at 1:1000; rabbit anti-PERK antibody (Cell Signaling Technology) was used for western blotting at 1:1000. Horseradish peroxidase-conjugated secondary antibodies were purchased from Rockland Immunochemicals and used at 1:5,000, and AlexaFluor secondary antibodies were purchased from Life Technologies and used at 1:500.

### Western blot analysis

Cells were lysed in NP40 lysis buffer (50 mM Tris pH 7.4, 150 mM NaCl, 0.5 mM EDTA, 0.5% NP40). Equal amounts of protein were separated by SDS-PAGE and transferred to a nitrocellulose membrane (Bio-Rad). Blocking was performed using 5% nonfat milk in Tris-buffered saline with 0.1% Tween 20 (TBST) for 1 hour at room temperature. Membranes were probed with the indicated primary and appropriate horseradish peroxidase-conjugated secondary antibodies. For detection of total and phosphorylated forms of proteins, samples were run in duplicate gels and transferred to independent membranes for western blotting. Membranes were probed for actin and protein levels were normalized in both membranes for the downstream densitometry analysis [26]. Proteins were detected using Western Lightning Plus-ECL (PerkinElmer). For quantitation, the pixel intensity of each band was determined using the ImageJ program (NIH) and then normalized to the indicated control.

### Indirect immunofluorescence

Cells were prepared for indirect immunofluorescence as described previously [27]. Briefly, cells were fixed in 4% paraformaldehyde and permeabilized with 0.2% Triton X-100. To prevent nonspecific binding, the cells were blocked using Roche Blocking Solution for 30 minutes at room temperature. Primary antibodies were applied followed by incubation with the appropriate secondary antibody in blocking solution. Stained cells were mounted in ProLong Gold Antifade Reagent with DAPI (Life Technologies). Laser scanning confocal microscopy was performed using a Leica DM16000B microscope equipped with a WaveFX spinning disk confocal head (Quorum Technologies) using a 40X objective lens. Images were acquired with a Hamamatsu ImageEM EM-charges coupled device (CCD) camera and collected as Z-stacks that were rendered for image reconstruction using the Imaris software (v. 8.1.3, Bitplane, Inc.).

### Quantification of stress granule assembly

Twenty-four hours after infection, Vero cells were treated with 500 μM Ars for 1 h, 2mM DTT for 1 h or 50 nM PatA for 1 h or U2OS cells were treated with 1 mM Se for 2 h and then processed for immunofluorescence as described above. Infected cells were identified by detection of viral protein NS1 or BrUTP labeled RNA, and SG-positive cells were defined as having at least 3 SG as determined by colocalized G3BP1 and TIAR or eIF4G and TIAR puncta. At least 150 cells were analyzed per condition in 10 to 15 fields in 3 independent experiments and the data are presented as the percentage of cells containing SG.

### Statistical analysis

All experiments were performed in triplicate, and the data are presented as the mean ± standard deviation (SD). A p-value <0.05 in a two-way ANOVA test was considered statistically significant. GraphPad Prism 6 (Graphpad Software Inc.) was used to conduct statistical analyses and create graphs.

## Results

### ZIKV recruits TIAR to sites of viral RNA replication

**Fig 1:**
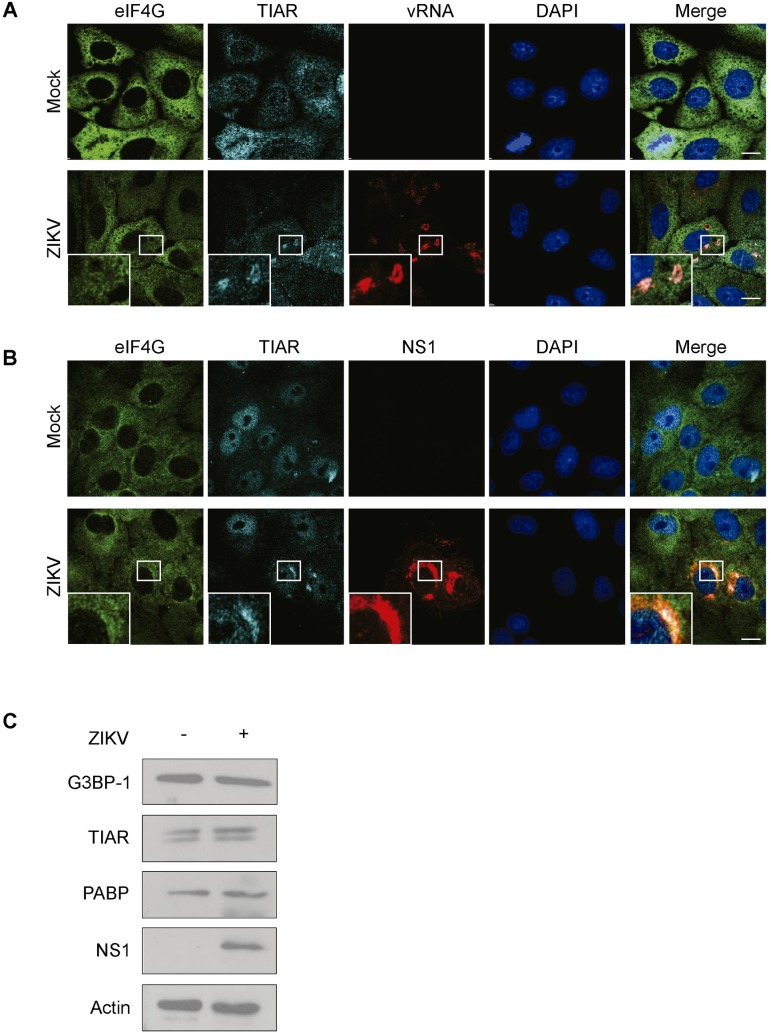
ZIKV recruits TIAR to sites of viral RNA replication. Vero cells were infected with ZIKV with an MOI of 0.5 and A. at 24 hpi, cells were treated with Actinomycin D and the nascent viral RNA was labeled with BrUTP and detected by immunofluorescence/laser scanning confocal microscopy (IF/LSCM) using a 40X objective lens; B. at 24 hpi, cells were fixed and the viral protein NS1 was detected by immunofluorescence followed by confocal microscopy. Representative of 2 experiments; C. at 24 hpi, cells were lysed and lysates were analyzed for G3BP-1, TIAR, PABP, NS1 and β-actin by SDS-PAGE followed by Western blotting.

ZIKV is a positive-strand RNA virus that replicates in the cytoplasm but little is known about redistribution of host proteins in ZIKV infected cells. Sequestering SG components to sites of viral replication is a strategy used by viruses to impair SG assembly in infected cells [18]. To determine whether ZIKV replication altered the distribution of SG markers, Vero cells were infected with ZIKV with an MOI of 0.5 and 6, 12 and 24 hours after, nascent viral RNA was labeled with BrUTP and detected by indirect immunofluorescence. In ZIKV-infected cells, viral protein or RNA was not detectable to 12 hpi. TIAR was evenly distributed throughout mock-infected cells (Fig 1A and 1B), and eIF4G was distributed homogeneously in the cytoplasm (Fig 1A and 1B). However, in infected cells, TIAR was still found in both the cytoplasm and nucleus but also concentrated in foci in the perinuclear region (Fig 1A and 1B), colocalizing with the ZIKV RNA (Fig 1A) and viral nonstructural protein, NS1 (Fig 1B). No change in eIF4G distribution was observed between mock and infected cells (Fig 1A and 1B). These findings suggest that ZIKV infection induces the redistribution of TIAR to sites of viral RNA replication.

Cleavage of proteins that nucleate SG assembly has been reported to be a strategy employed by viruses to overcome cellular stress response [28]. We next evaluated whether ZIKV replication alters the levels of SG markers. Cells were mock infected or infected with ZIKV and at 24 hpi cells lysates were collected and analyzed by SDS-PAGE followed by Western blotting. No alteration was observed in the levels of G3BP-1, TIAR and PABP between mock and infected cells (Fig 1C). These results indicate that ZIKV infection does not induce changes in the levels of SG-nucleating proteins.

### ZIKV infection blocks SG assembly triggered by arsenite treatment

**Fig 2:**
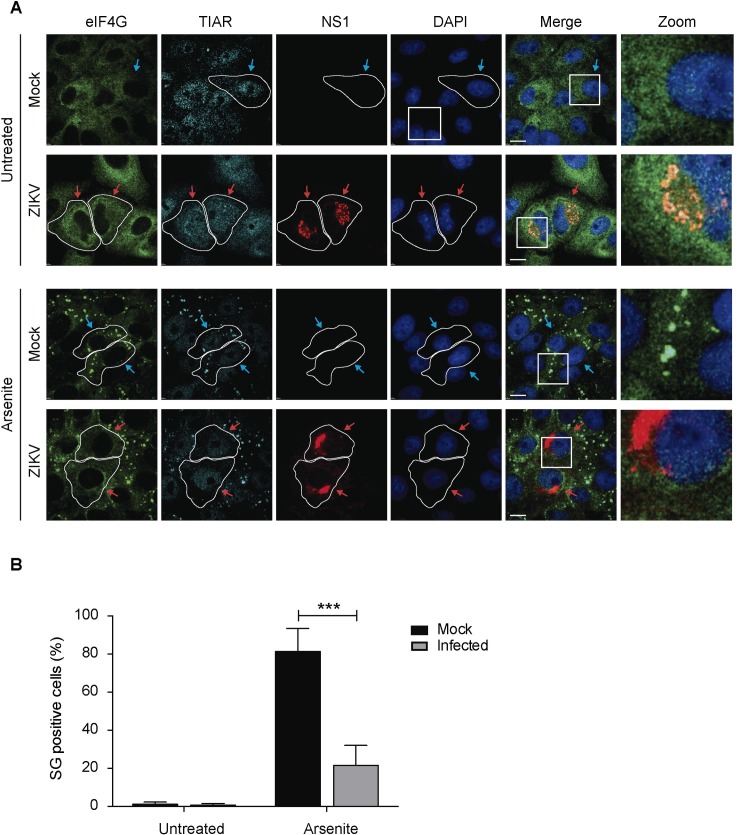
ZIKV infection blocks Ars-induced SG assembly. A. Vero cells were infected with ZIKV with an MOI of 0.5 or mock-infected and treated at 24 hpi with 500 μM Ars for 1 h to induce cellular stress. The SG markers TIAR and eIF4G were observed by IF/LSCM and infected cells were identified by the presence of the viral protein NS1. Blue arrows: uninfected cells; red arrows: infected cells. B. At least 150 cells in each condition were analyzed. Cells with at least 3 SG were considered positive. Data are presented as mean ± SD from 3 independent experiments.

Several viruses, including many members of Flaviviridae family, have the ability to modulate SG assembly to keep the cell environment favorable to their own replication [29, 30]. We investigated whether ZIKV can interfere with the assembly of SG in infected cells. Vero cells were infected with ZIKV or mock-infected and treated with sodium arsenite (Ars) at 24 hours post-infection to induce cellular stress. Ars is an oxidative agent that rapidly induces SG assembly through phosphorylation of eIF2α [31]. SG assembly was determined by indirect immunofluorescence of TIAR and eIF4G and infected cells were identified by the presence of the viral protein NS1. In the absence of stress, mock-infected (blue arrows) and ZIKV-infected cells (red arrows) exhibited SG assembly at a rate of 0.61% and 0%, respectively (Fig 2A, top panels and Fig 2B), indicating that ZIKV infection does not induce the assembly of SG. In mock-infected cells, Ars treatment induced abundant SG assembly as expected, with 81.4% of the cells presenting TIAR and eIF4G co-localized in cytoplasmic puncta (Fig 2A and 2B). In contrast, ZIKV infected cells presented SG at a rate of only 21.6% (Fig 2A, bottom panel and Fig 2B). Similar results were observed when U2OS cells were used in place of Vero cells (S1 Fig). These results indicate that ZIKV infection blocks the assembly of type I SGs.

### ZIKV does not block eIF2α-independent assembly of stress granules

**Fig 3:**
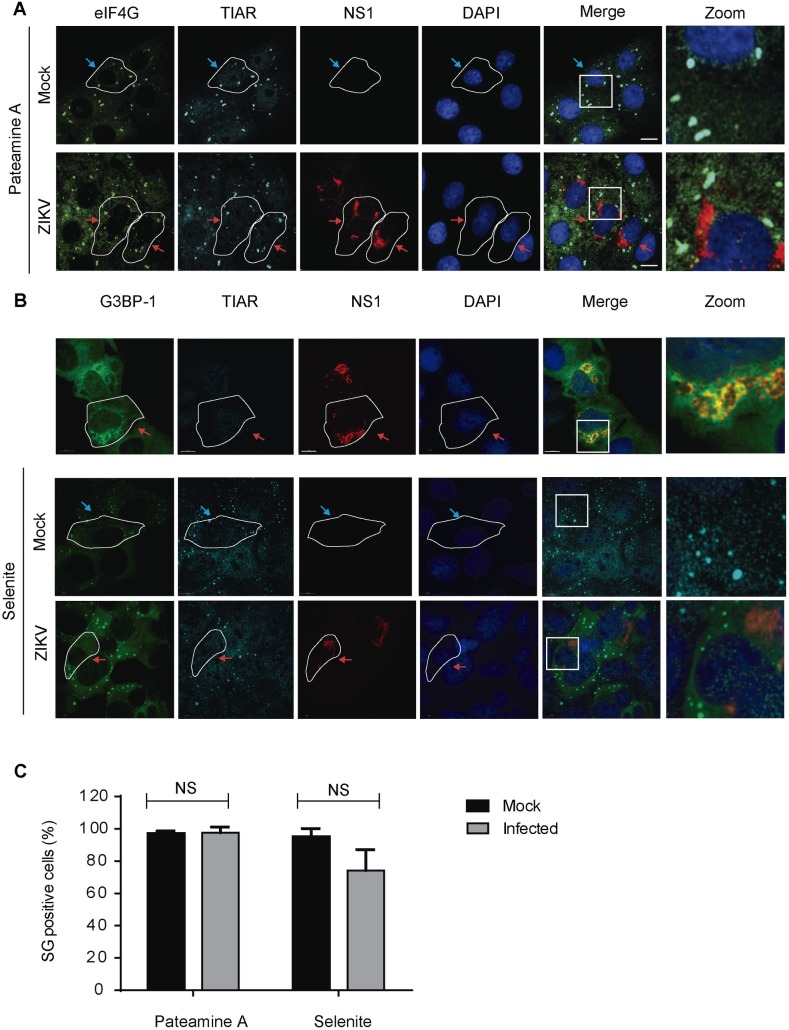
ZIKV does not block Se- or PatA-induced SG assembly. A. Vero cells were infected with ZIKV with an MOI of 0.5 or mock-infected and treated at 24 hpi with 50 nM PatA for 2 h to induce cellular stress. B. U2OS GFP-G3BP1-expressing cells were infected with ZIKV or mock-infected and treated at 24 hpi with 1 mM Se for 2 hours. SG assembly was determined by IF/LSCM staining for the SG markers TIAR and eIF4G (Vero cells) or G3BP-1 and TIAR (U2OS cells) and infected cells were identified by the presence of the viral protein NS1. Blue arrows: uninfected cells; red arrows: infected cells. C. At least 150 cells in each condition were analyzed. Cells with at least 3 SG were considered positive. Data are presented as mean ± SD from 3 independent experiments.

Pateamine A (PatA) is a natural product isolated from a marine sponge that disrupts the translation initiation by hyperactivating the eIF4A helicase and disrupting the eIF4F complex, leading to the assembly of SG in an eIF2α-independent manner [32]. To test whether ZIKV infection was also capable of blocking PatA-induced SGs, Vero cells were mock-infected or infected with ZIKV and at 24 hpi were treated with PatA. SG assembly was determined by colocalized puncta of TIAR and eIF4G and infected cells were identified by the presence of the viral protein NS1. PatA treatment, as expected, induced a robust SG assembly in 97.2% of the mock-infected cells (Fig 3A, top panels, and 3B). Interestingly, ZIKV infection did not impair PatA-induced SG assembly, as 97.5% of the infected cells presented TIAR and eIF4G puncta (Fig 3A, top panels, and 3B).

Sodium selenite (Se) promotes the assembly of type II SG that differ from canonical SGs in their morphology, composition and mechanism of assembly, mainly by disrupting the eIF4F complex formation through 4EBP1 [33]. To test whether ZIKV infection alters Se-induced SG assembly, U2OS cells (S1 Fig) stably expressing GFP-G3BP1 were mock-infected or infected with ZIKV and at 24 hpi were treated with Se. U2OS cells were used in place of Vero cells due to the high toxicity of Se to the latter ones. SG assembly was determined by colocalized puncta of TIAR and G3BP-1 and infected cells were identified by the presence of the viral protein NS1. Similarly to PatA-induced SG, no significant difference was observed in the assembly of SG between mock and infected cells treated with Se (Fig 3A, bottom panels, and 3B). These findings indicate that ZIKV infection blockage of SG assembly is eIF2α-dependent.

### ZIKV blocks eIF2α phosphorylation triggered by arsenite

**Fig 4:**
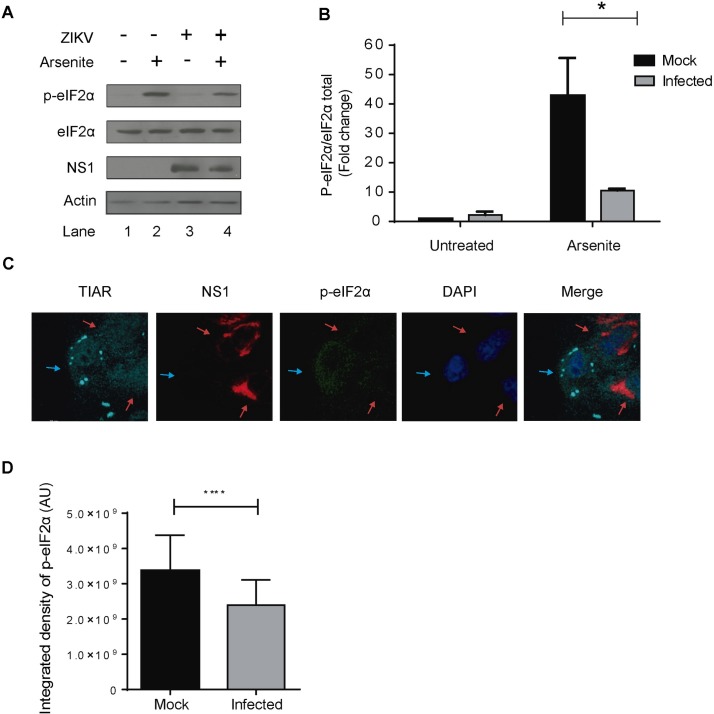
ZIKV blocks Ars-induced eIF2α phosphorylation. A. Vero cells were infected with ZIKV with an MOI of 0.5 or mock-infected and treated at 24 hpi with 500 μM Ars for 1 h to induce cellular stress. Lysates were analyzed for S51-phospho(P)-eIF2α, eIF2α (total) and NS1 by SDS-PAGE followed by Western blotting. B. Densitometry quantification of p-eIF2α was determined by ImageJ analysis. Values presented in the graph are normalized against the total amount of eIF2α in the cell lysate and represent fold change with the untreated mock-infected cells being arbitrarily set to 1. Asterisks represent the statistically significant difference between mock and ZIKV-infected cells (Two-way ANOVA; p < 0.05). C. Cellular stress was determined by IF/LSCM staining for SG markers, TIAR and phosphor-eIF2α and infected cells were identified by the presence of the NS1 viral protein. Blue arrows: uninfected cells; red arrows; infected cells. D. Quantification of the integrated density of p-eIF2α signal by ImageJ analysis.

Many viruses modulate p-eIF2α levels during replication to assure viral protein synthesis and avoid cellular stress responses. For example, coronaviruses can induce GADD34 expression to enhance PP1 activity and consequently the dephosphorylation of eIF2α [34], and herpesviruses encode a viral protein that mimics the function of GADD34 [35]. We examined the phosphorylation status of eIF2α in ZIKV infected untreated or Ars treated cells. Protein lysates were analyzed by Western blotting using an antibody specific for eIF2α phosphorylation at S51. As shown in Fig 4A and 4B, little phosphorylation of eIF2α was detected in mock-infected and untreated Vero cells, with a slight increase in p-eIF2α in ZIKV-infected cells. As expected, high levels of eIF2α phosphorylation (40-fold increase) were observed in extracts of mock-infected cells treated with Ars. However, in ZIKV-infected and Ars treated cells, levels of eIF2α phosphorylation were consistently lower (10.5-fold increase). ZIKV replication was confirmed by the detection of the viral protein NS1 in cell extracts. The amount of total eIF2α was similar under all conditions tested (Fig 4A), indicating that ZIKV replication does not alter its expression. To further confirm that ZIKV-infected cells exhibit lower levels of p-eIF2α under arsenite treatment, phosphorylation of eIF2α was also analyzed by IF/LSCM. As shown in Fig 4C, phosphorylation of eIF2α is strongly induced in the cytoplasm of non-infected cells (blue arrows). In contrast, in ZIKV-infected cells, the phospho-eIF2α signal is visibly weaker (red arrow). Upon arsenite treatment, the fluorescence intensity of p-eIF2α in infected cells was in average 30% lower in ZIKV-infected cells in comparison to mock-infected cells (Fig 4D). These results indicate that ZIKV infection impairs eIF2α phosphorylation triggered by oxidative stress.

### ZIKV blocks SG assembly and eIF2α phosphorylation triggered by ER stress

**Fig 5:**
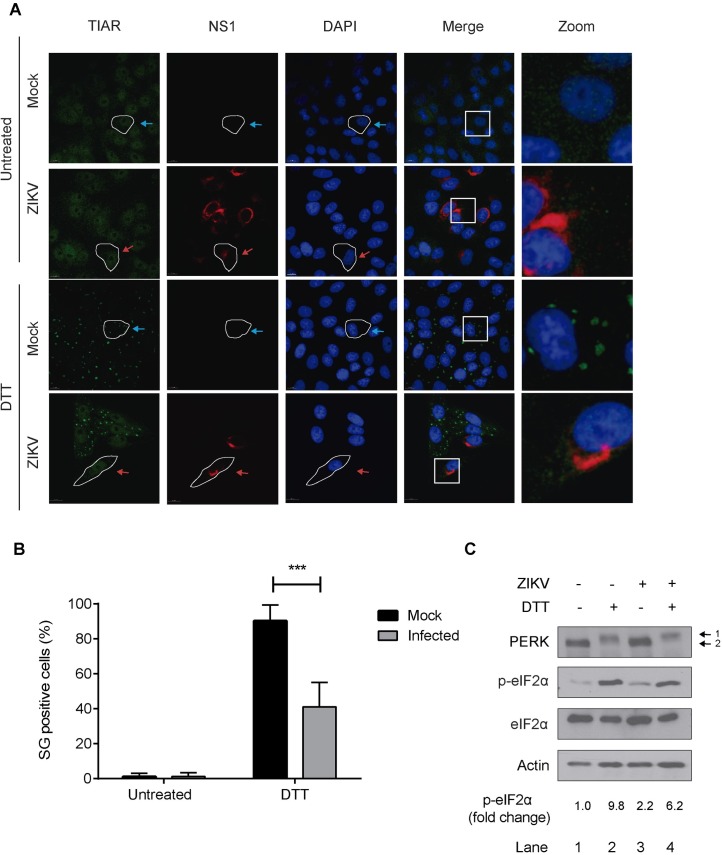
ZIKV infection blocks DTT-induced SG assembly and phosphorylation of eIF2α. A. Vero cells were infected with ZIKV with an MOI of 0.5 or mock-infected and treated at 24 hpi with 2mM DTT for 1 h to induce cellular stress. The SG marker TIAR was observed by IF/LSCM and infected cells were identified by the presence of NS1. Blue arrows: uninfected cells; red arrows: infected cells. B. At least 150 cells in each condition were analyzed. Cells with at least 3 SG were considered positive. Data are presented as mean ± SD from 3 independent experiments. C. After DTT treatment, cells were lysed and lysates were analyzed for PERK, S51-phospho(P)-eIF2α, eIF2α (total) and actin by SDS-PAGE followed by Western blotting. Densitometry quantification of p-eIF2α was determined by ImageJ analysis. Values presented are normalized against the total amount of eIF2α in the cell lysate and represent fold change with the untreated mock-infected cells being arbitrarily set to 1.

Our findings show that ZIKV infection blocks SG assembly and phosphorylation of eIF2α triggered by Ars, an HRI activator. To investigate whether this blockage is dependent on the eIF2α kinase activated upon stress, Vero cells were mock-infected or infected with ZIKV and at 24 hpi were treated with DTT, an endoplasmic reticulum (ER) stressor that activates PERK. SG assembly was determined by TIAR puncta and infected cells were identified by the presence of NS1. DTT treatment induced SG assembly in 81.8% of the mock-infected cells (Fig 5A and 5B). In contrast, only 28.6% of ZIKV-infected cells presented SG (Fig 5A and 5B). The blockage of SG assembly correlates with lower levels of p-eIF2α upon DTT treated in ZIKV-infected cells (Fig 5C, lane 4) when compared to mock-infected cells (Fig 5C, lane 2). Interestingly, the activation of PERK in DTT-treated cells, demonstrated by an increased PERK mobility, was similar in mock and ZIKV-infected cells (Fig 5C, compare lanes 2 and 4, position 1: activate PERK; position 2: inactive PERK), suggesting that the reduced levels of p-eIF2α in infected cells are a result of an interference downstream the activation of the eIF2α kinases.

### eIF2α dephosphorylation is modulated by ZIKV

**Fig 6:**
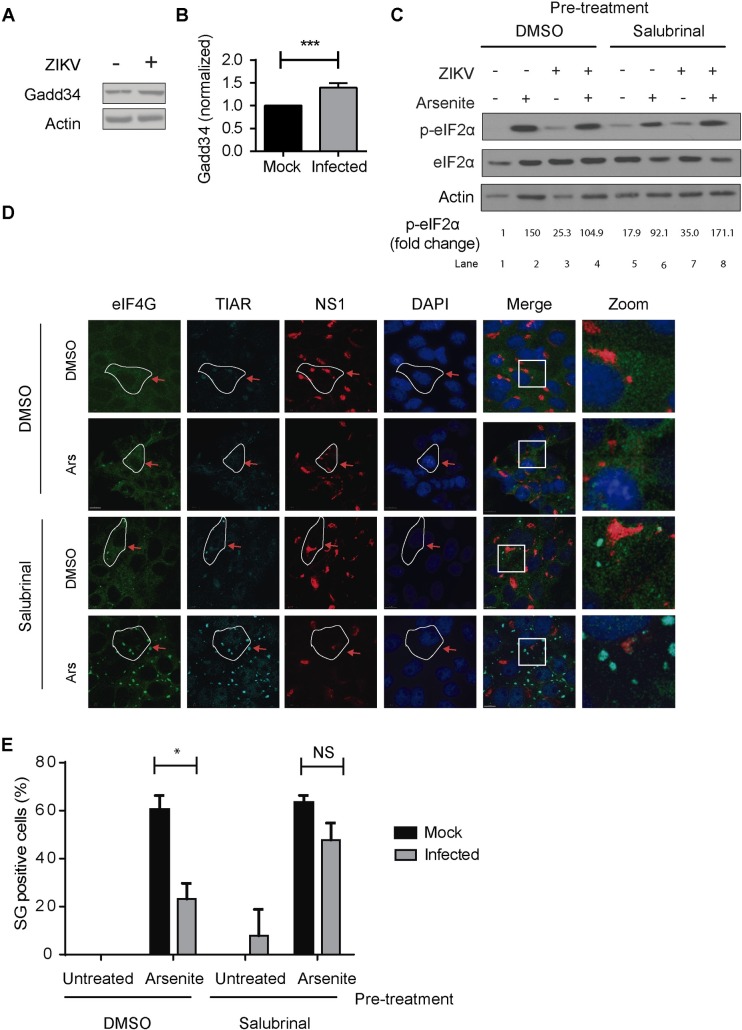
ZIKV modulates eIF2α dephosphorylation. A. Vero cells were infected with ZIKV at an MOI of 0.5 or mock-infected. At 24 hpi, cells were lysed and cells were lysed and lysates were analyzed for GADD34 and actin by SDS-PAGE followed by Western blotting. B. Densitometry quantification of GADD34 and actin were determined by ImageJ analysis. Values presented are normalized against the total amount of GADD34 in the cell lysate and represent fold change with the mock-infected cells being arbitrarily set to 1. C. Vero cells were infected with ZIKV or mock-infected and treated at 24 hpi with 75 μM salubrinal for 3 h to block the dephosphorylation of eIF2α and then treated with 500 μM Ars for 1 h to induce cellular stress. Lysates were analyzed for S51-phospho(P)-eIF2α and eIF2α (total) by SDS-PAGE followed by Western blotting. Values of p-eIF2α fold change were normalized by the corresponding eIF2α levels of the same condition. D. Vero cells were infected with ZIKV or mock-infected and at 24 hpi were treated with 75 μM salubrinal for 3 h and then oxidative stress was induced by treatment with 500 μM Ars for 1 h. SG assembly was determined by IF/LSCM staining for the SG markers TIAR and eIF4G and infected cells were identified by the presence of the viral protein NS1. Blue arrows: uninfected cells; red arrows: infected cells.E. At least 150 cells in each condition were analyzed. Cells with at least 3 SG were considered positive. Data are presented as mean ± SD from 3 independent experiments and asterisks represent the statistically significant difference between mock and ZIKV-infected cells (Two-way ANOVA; p < 0.05).

Our results suggest that ZIKV infection might abrogate SG assembly by blocking eIF2α phosphorylation. To test this further, Vero cells were infected with ZIKV and at 24 hpi, the levels of GADD34, a PP1A cofactor, were evaluated. Our results show that are GADD34 levels are significantly higher in ZIKV-infected cells as compared to uninfected cells (Fig 6A and 6B). To evaluate the role of GADD34/PP1A activity on ZIKV-infected cells, we treated cells with salubrinal and its derivative sal003, small molecules that selectively inhibit the PP1/GADD34-mediated dephosphorylation of phospho-eIF2α [36, 37]. Vero cells were treated with 75 μM of salubrinal or 10 μM of sal003 for 3 h prior to the addition of Ars to the cells. The phosphorylation status of eIF2α was evaluated by western blotting analysis (Fig 6C). In cells treated with salubrinal prior to Ars-induced stress, ZIKV-infected cells present higher levels of phospho-eIF2α as compared to mock-infected control (Fig 6C, compare lanes 6 and 8). Similar results were obtained with sal003 [37] (S2 Fig). The assembly of SG in the distinct conditions was monitored by indirect immunofluorescence. SGs were induced in 23.1±6.5% of ZIKV-infected cells treated with Ars. This value increased to 47.8±7.0% in cells that were treated with salubrinal prior to Ars-induced stress and was not significantly different from mock-infected cells (Fig 6D and 6E). No significant difference was observed between control or salubrinal pre-treated mock-infected cells (Fig 6E). Hence, inhibiting eIF2α dephosphorylation reduces the ability of ZIKV infection to block Ars-induced SG assembly. These results indicate that eIF2α dephosphorylation is differentially modulated during ZIKV replication and that this feature can contribute to ZIKV-mediated blockage of SG assembly.

### Salubrinal inhibits ZIKV replication

**Fig 7:**
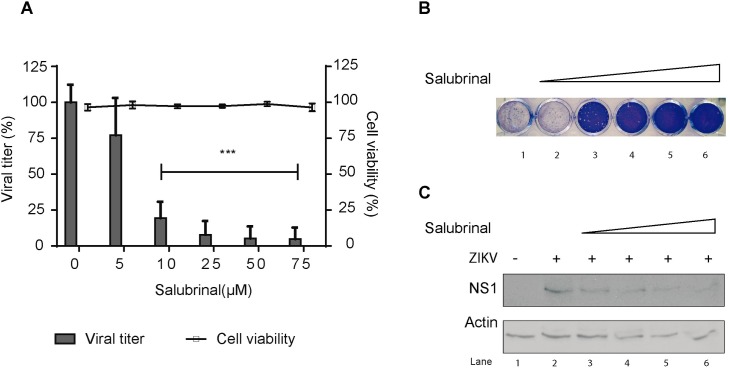
Salubrinal inhibits ZIKV replication. Vero cells were infected with ZIKV at an MOI of 0.5 or mock-infected and after 1 h of adsorption, cells were incubated with 5 to 75 μM salubrinal. Cells were incubated for 24 h and A. supernatants of each condition were collected and viral titer was determined by plaque forming assay (bar graph) and cell viability of mock-infected cells was determined by trypan blue exclusion (line graph); B. representative result of the plaque forming assay in A; C. Cell lysates were analyzed for NS1 and β-actin by SDS-PAGE followed by Western blotting.

To further confirm the importance of modulating eIF2α for ZIKV replication, Vero cells were infected with ZIKV and after 1 hpi, salubrinal was added to culture media in increasing concentrations. After 24 h, supernatants of each condition were collected and viral titer was determined by plaque forming assay and cells were lysed and lysates were processed by SDS-PAGE followed by Western blotting. Treatment of cells with salubrinal led to a dose-dependent decrease in the production of infectious particles released to the culture media (Fig 7A, bar graph and 7B). Cells treated with 75 μM of salubrinal produce only 4.9% of the infectious viral particles produced by control cells (Fig 7A, bar graph and 7B). Salubrinal had no toxic effects on treated cells (Fig 7A, line graph). Finally, a dose-dependent decrease in NS1 expression was observed in salubrinal treated cells (Fig 7C).

## Discussion

The relationship between viruses and the cellular stress response is a multifaceted and complex phenomenon that depends on the structural and genetic characteristics of the virus and the host cell [38]. Infection by several types of RNA and DNA viruses results in changes in the cellular environment as viral replication co-opts several cellular pathways, including nutrient, energy and macromolecular synthesis, to produce infectious particles. In this process, viruses trigger the host cell stress response, which can lead to the assembly of SGs [17]. Since viral replication relies on the host translational machinery, most viruses suppress the stress response pathway and SG assembly at some point of their replicative cycle [18]. Interactions between stress proteins and viral components have been described in a large variety of experimental models at different stages of the viral lifecycle, depending on the type of virus and host cell [29, 39].

ZIKV has emerged as a global public health threat over the last decade. Many aspects of the molecular mechanisms involved in the pathogenesis of this emerging virus remain unclear and require further investigation. In this work, we described that ZIKV replication does not induce SG assembly in Vero cells (Fig 1). This contrasts with the results recently published by Roth and colleagues [19] that describe the assembly of SG-like structures on Huh-7 cells infected with ZIKV. It is possible that those distinct findings are due to the usage of distinct cell lines. In our work, we also show that ZIKV and blocks SG assembly triggered by treatment of cells with Ars (Fig 2) and DTT (Fig 5). These finds are similar to the ones described recently by Roth and colleagues, in which they describe that flaviviruses block SG assembly independently of the eIF2α kinase activated by stress [19]. Interestingly, during the review process of this manuscript, Basu and colleagues [40] reported that ZIKV-mediated blockage of SG assembly was specific for oxidative stress induced by arsenite. The reasons why these differences were observed remain to be determined. Several reports have shown that members of the Flaviviridae family modulate SG assembly in infected cells. The 3’ stem loop from the viral minus strand of WNV and DENV captures TIA-1 and TIAR to promote viral genome RNA synthesis and inhibit SG assembly [29, 41]. JEV capsid protein interaction with Caprin-1 leads to the sequestration of several SG components, such as G3BP1 and USP10, in the perinuclear region of infected cells, resulting in impairment of SG assembly [30] and bovine viral diarrhea virus (BVDV) blocks Ars-mediated SG assembly [42]. TIA1 and TIAR are recruited to tick-borne encephalitis virus (TBEV) sites of replication [43]. Finally, hepatitis C virus (HCV) replication leads to oscillating SG assembly/disassembly in infected cells through controlling the phosphorylation of eIF2α and co-opting TIA-1, TIAR and G3BP1 [44, 45]. More recently, Roth and colleagues [19] demonstrated that DENV and ZIKV uncouple translation suppression from the stress response by a mechanism that is yet to be identified.

We demonstrate that ZIKV infection did not lead to a blockage in PatA or Se-induced SG (Fig 3). The assembly of SG triggered by both molecules is independent of the phosphorylation of eIF2α, suggesting that ZIKV blocks stress granules assembly mainly via eIF2α signaling. Interestingly, this does not seem to be a general feature of flaviviral infections, as it has been demonstrated by Roth and colleagues that DENV inhibits SG assembly induced by hippuristanol, an inhibitor of eIF4A RNA binding [19]. The phosphorylation of eIF2α is a key regulator of mRNA translation initiation, and the level of phospho-eIF2α is modulated by the activities of kinases and phosphatases [46]. Oxidative stress induced by Ars culminates on eIF2α phosphorylation by HRI [31], which prevents the recycling of the eIF2-GTP-tRNA^Met^ ternary complex, leading to polysome disassembly and consequent translational arrest and SG assembly [14].

Regulation of protein synthesis by eIF2α phosphorylation plays an important role in the cellular defense against viral infection, thus viruses evolved diverse strategies to prevent it. Our results show that ZIKV attenuates eIF2α phosphorylation triggered by Ars (Fig 4) and DTT (Fig 5) and this ability is, at least in part, a consequence of modulating its dephosphorylation, as supported by the observation that treatment of cells with salubrinal reverses the ZIKV-mediated blockage of SG assembly induced by Ars (Fig 6). Similar to the finding of Wang and colleagues using coronavirus [34], we demonstrated that ZIKV infection induces a moderate increase in GADD34 expression (Fig 6A and 6B). Recently, Buchman and colleagues [47] described a mechanism by which trehalose modulates p-eIF2α levels and stress granule assembly/disassembly by enhancing the expression of GADD34 and CReP. The increase in the cellular levels of the PP1 phosphatase subunits could lead to faster dephosphorylation of p-eIF2α and disassembly of SGs, thereby rendering the cells able to recover more quickly from stress. It is possible that the enhanced levels of GADD34 found in ZIKV-infected cells play a similar role in response to stress.

Treatment of cells with salubrinal causes an accumulation of phospho-eIF2α through an inhibition of PP1/GADD34-mediated dephosphorylation of eIF2α without increasing eIF2α kinase activity [36]. Modulation of PP1 activity by viral infection was demonstrated for human cytomegalovirus [48], African swine fever virus [49], Newcastle disease virus [50], papillomavirus [51] and herpes simplex virus [52]. ICP34.5 is a protein homologous to GADD34 encoded by HSV that is essential for HSV replication in some cell types. It binds cellular PP1 and promotes eIF2α dephosphorylation, ensuring viral replication despite activation of PKR [35]. Treatment of HSV-infected cells with salubrinal inhibits viral replication in a dose-dependent manner and leads to higher phospho-eIF2α levels [36, 52]. Similarly, our results demonstrate that ZIKV replication is severely impaired in salubrinal-treated cells (Fig 7), indicating that ZIKV relies to some extent on eIF2α dephosphorylation for its replication. These findings are distinct from the model proposed by Roth and colleagues [19], in which modulation of SG assembly in ZIKV-infected cells was independent of eIF2α dephosphorylation promoted by elevated GADD34 levels. Cell type-specificities can be responsible for those contrasting results. It remains to be determined whether treatment with salubrinal has secondary effects on the infected cells that could act in synergy with the PP1/GADD34 inhibition and the mechanism by which ZIKV modulates this activity.

In conclusion, our work provides new insights into the ZIKV biology by demonstrating that ZIKV inhibits SG assembly in a phospho-eIF2α dependent way. This ability may reflect one of the many strategies that ZIKV has evolved to control the host stress response and demonstrate that ZIKV elicits mechanisms to counteract host anti-viral stress responses to promote a cellular environment propitious for viral replication. Elucidation of the interaction of viral components with host factors involved in SG assembly may provide new insights into the pathology of ZIKV infection and lead to the identification of novel targets for therapeutic intervention.

## Supporting information

S1 Fig ZIKV infection blocks Ars-induced SG assembly in U2OS cells. A. U2OS cells were infected with ZIKV with an MOI of 0.5 or mock-infected and treated at 24 hpi with 500 μM Ars for 1 h to induce cellular stress. The SG markers G3BP-1 and eIF4G were observed by IF/LSCM and infected cells were identified by the presence of the viral protein NS1. Blue arrows: uninfected cells; red arrows: infected cells. B. At least 150 cells in each condition were analyzed. Cells with at least 3 SG were considered positive. Data are presented as mean ± SD from 3 independent experiments. C. U2OS cells were infected with ZIKV with an MOI of 0.5 or mock-infected and treated at 24 hpi with 500 μM Ars for 1 h to induce cellular stress. Lysates were analyzed for S51-phospho(P)-eIF2α and eIF2α (total) by SDS-PAGE followed by Western blotting. B. Densitometry quantification of p-eIF2α was determined by ImageJ analysis. Values presented in the graph are normalized against the total amount of eIF2α in the cell lysate and represent fold change with the untreated mock-infected cells being arbitrarily set to 1. Asterisks represent the statistically significant difference between mock and ZIKV-infected cells (Two-way ANOVA; p < 0.05)
(TIF)

S2 Fig eIF2α dephosphorylation modulated by ZIKV is inhibited by sal003. Vero cells were infected with ZIKV or mock-infected and treated at 24 hpi with 10 μM sal003 for 3 h to block the dephosphorylation of eIF2α and then treated with 500 μM Ars for 1 h to induce cellular stress. Lysates were analyzed for S51-phospho(P)-eIF2α and eIF2α (total) by SDS-PAGE followed by Western blotting. Values of p-eIF2α fold change were normalized by the corresponding eIF2α levels of the same condition.
(TIF)
