# Supplementary figures and images for: Zika virus inhibits eIF2α-dependent stress granule assembly
Source: PLoS Negl Trop Dis. 2017 Jul 17;11(7):e0005775. doi: 10.1371/journal.pntd.0005775 (PMC5531678; doi:10.1371/journal.pntd.0005775)

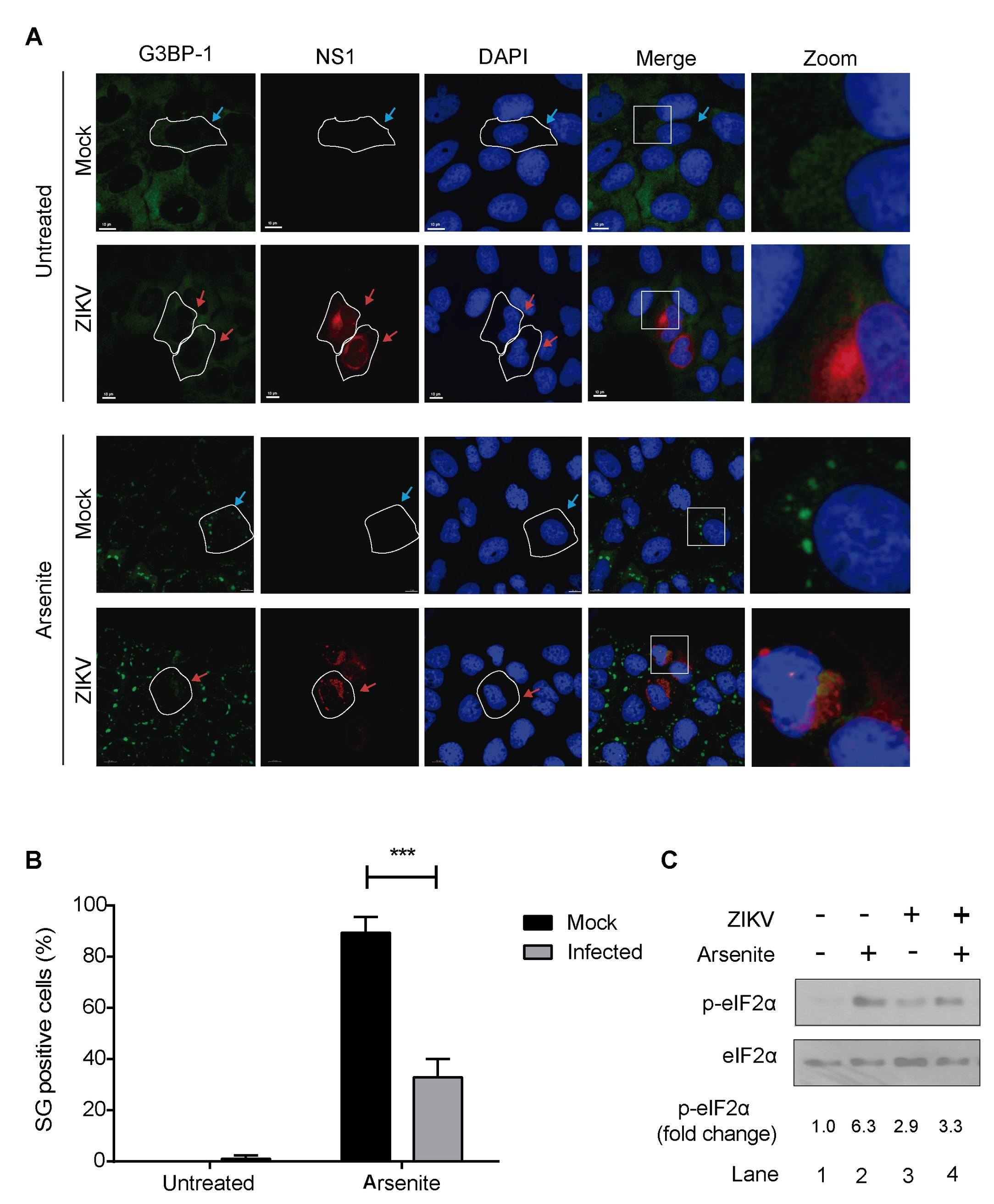

Supplement: S1 Fig — A. U2OS cells were infected with ZIKV with an MOI of 0.5 or mock-infected and treated at 24 hpi with 500 μM Ars for 1 h to induce cellular stress. The SG markers G3BP-1 and eIF4G were observed by IF/LSCM and infected cells were identified by the presence of the viral protein NS1. Blue arrows: uninfected cells; red arrows: infected cells. B. At least 150 cells in each condition were analyzed. Cells with at least 3 SG were considered positive. Data are presented as mean ± SD from 3 independent experiments. C. U2OS cells were infected with ZIKV with an MOI of 0.5 or mock-infected and treated at 24 hpi with 500 μM Ars for 1 h to induce cellular stress. Lysates were analyzed for S51-phospho(P)-eIF2α and eIF2α (total) by SDS-PAGE followed by Western blotting. B. Densitometry quantification of p-eIF2α was determined by ImageJ analysis. Values presented in the graph are normalized against the total amount of eIF2α in the cell lysate and represent fold change with the untreated mock-infected cells being arbitrarily set to 1. Asterisks represent the statistically significant difference between mock and ZIKV-infected cells (Two-way ANOVA; p < 0.05) (TIF) [file pntd.0005775.s001.tif]

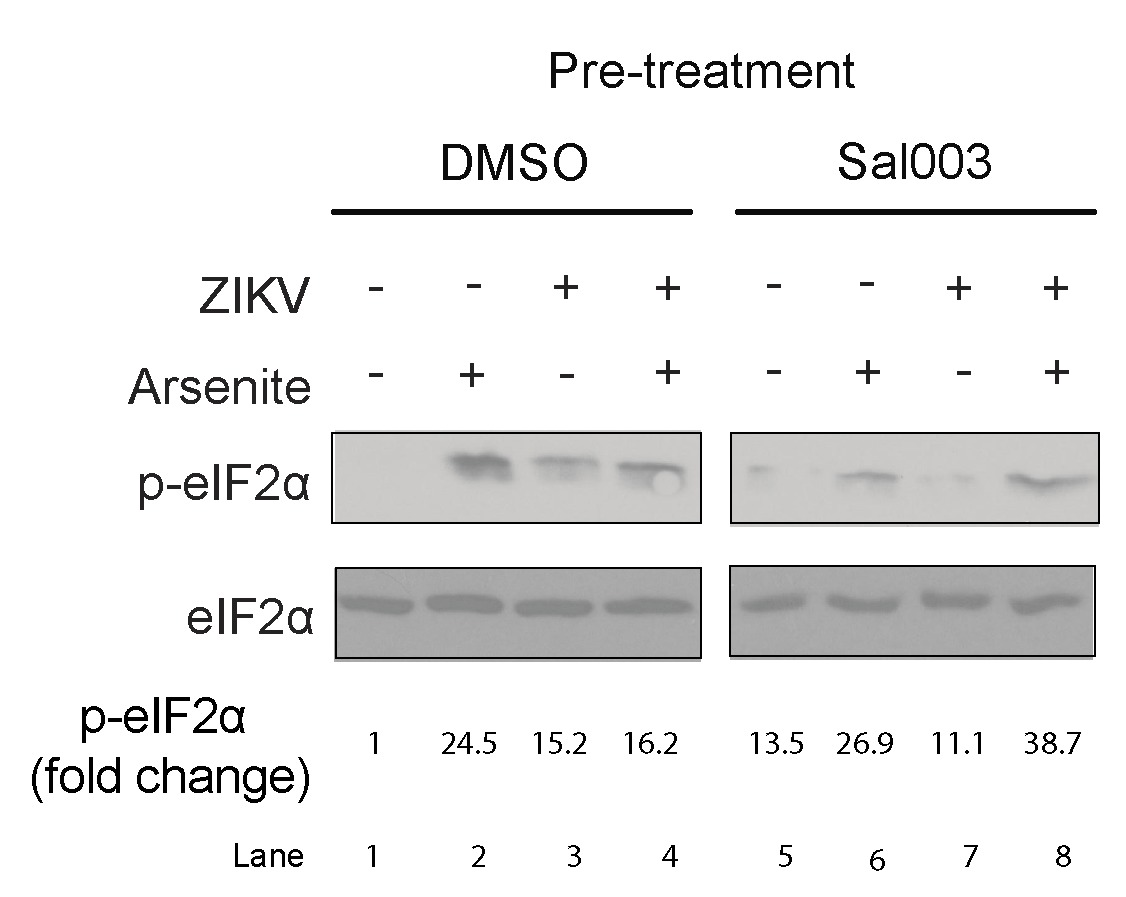

Supplement: S2 Fig — Vero cells were infected with ZIKV or mock-infected and treated at 24 hpi with 10 μM sal003 for 3 h to block the dephosphorylation of eIF2α and then treated with 500 μM Ars for 1 h to induce cellular stress. Lysates were analyzed for S51-phospho(P)-eIF2α and eIF2α (total) by SDS-PAGE followed by Western blotting. Values of p-eIF2α fold change were normalized by the corresponding eIF2α levels of the same condition. (TIF) [file pntd.0005775.s002.tif]
